# The Lay-Medical Dinner

**Published:** 1913-11-29

**Authors:** 


### THE LAY-MEDICAL DINNER.

Opportunities for doctors to exchange ideas with laymen on neutral ground, somewhere away from the sick-chamber or the consulting-room, are too rare in this country. We should open our eyes wide if the *Daily Mail* feuilleton consisted of very frank disquisitions by a professor of medicine on the medical aspects of all sorts of matters of common interest, who signed his name at the end, or rather the beginning, of his remarks, and occupied a considerable part of the correspondence columns with the discussions raised thereby. But, then, Germany is the land of intellectual authority, which England (to its loss, in spite of Meredith) is not. Something, however, may be done if one strokes the national temperament the right way. The public dinner is among the deepest rooted of national institutions. Therefore let the doctors and the not-doctors (to make a complete dichotomy of it) come together at public dinners, where they will not be in the relation of employers and employed, or have any other interest to divide them, and when the close of day encourages confidences.

But this expedient, it may be said, has been hit upon already times without number. So it has; but not very often with the particular variation which, as the result of an event last week, we have in mind. Granted the toast of "Medicine" has been proposed often enough at convivial gatherings, and as often responded to by a heavy senior in the time-honoured way, and that the ordinary medical officer of health does a good deal of dining out: nothing very much is effected in either case. In the one, the lump is too big for the not particularly active bit of leaven—which tends to be used again and again, the taste for oratory not being common in the profession; and in the other, there is that already mentioned factor, the relation of employer and employee, which congeals the flow of self-expression at its source. But if you arrange matters the reverse way, and bring your leading laymen to your medical dinner, the prospect becomes more interesting. Will they come, however? In one instance, at least, that question was answered clearly enough, when last week the Newcastle Clinical Society, mustering 140 strong of medical men, had on its short toast list four speakers from the laity, among these being the Lord Mayor and the classical professor from the local college. The occasion does not matter just now, being of technical interest, but the function was one which George Gissing, that able and sympathetic portrayer of provincial academic life, would have welcomed as a subject. The President, Dr. Patterson, after disposing of his main theme, made it clear that a spirit of intimacy between the civic and the medical life of the city was in favour with the society. The idea is excellent, as also, for a beginning, the means used to realise it. One does not need to labour such a commonplace as the rapidly growing importance to the community of the medical profession. Even clerics themselves will own politely, if a little sadly, that they are being supplanted as regards their former position of influence. And something like the ancient symposium promotes the diffusion and exchange of knowledge much better than didactic writing or speaking. Most men are fond of a well-conducted informal discussion, and all much more influenced by things they observe and conclusions they draw for themselves than by what is rubbed into them. A dinner would not frighten good practical-minded folk who would think congresses and conferences out of their line or over their heads. We hope, in short, to see such dinners held elsewhere, and believe there is only one needful precaution to be borne in mind, which is for the old set style of after-dinner oratory to be banished. The penalty to be meted out to the emitter of platitudes must be prompt suppression, or at any rate future exclusion.

